# Investigating the Hepatic Response to Orlistat and White Tea in Rats on a High-Fat Diet

**DOI:** 10.3390/life14101283

**Published:** 2024-10-10

**Authors:** Serpil Ciftel, Aleksandra Klisic, Enver Ciftel, Tolga Mercantepe, Adnan Yilmaz, Sedat Ciftel, Esra Pinarbas, Merve Nur Toraman, Filiz Mercantepe

**Affiliations:** 1 Department of Endocrinology and Metabolism, Erzurum Training and Research Hospital, 25100 Erzurum, Turkey; serpil.ciftel@saglik.gov.tr; 2 Faculty of Medicine, University of Montenegro, 81000 Podgorica, Montenegro; aleksandranklisic@gmail.com; 3 Center for Laboratory Diagnostics, Primary Health Care Center, 81000 Podgorica, Montenegro; 4 Department of Endocrinology and Metabolism, Sivas Numune Hospital, 58060 Sivas, Turkey; enver.ciftel@saglik.gov.tr; 5 Department of Histology and Embryology, Faculty of Medicine, Recep Tayyip Erdogan University, 53100 Rize, Turkey; 6 Department of Biochemistry, Faculty of Medicine, Recep Tayyip Erdogan University, 53100 Rize, Turkey; adnan.yilmaz@erdogan.edu.tr (A.Y.); esra.pinarbas@erdogan.edu.tr (E.P.); 7 Department of Gastroenterology and Hepatology, Erzurum Training and Research Hospital, 25100 Erzurum, Turkey; sedat.ciftel@saglik.gov.tr; 8 Department of Nutrition and Dietary, Faculty of Medicine, Recep Tayyip Erdogan University, 53100 Rize, Turkey; mervenur.toraman@saglik.gov.tr; 9 Department of Endocrinology and Metabolism, Faculty of Medicine, Recep Tayyip Erdogan University, 53100 Rize, Turkey

**Keywords:** hepatosteatosis, high-fat diet, liver, Nf-kB/p65, rat, white tea

## Abstract

High-fat diets have detrimental health impacts that increase the likelihood of developing obesity and metabolic syndrome. This study aimed to examine the potential antioxidant and anti-inflammatory effects of orlistat and white tea in rats fed a high-fat diet. Thirty-two rats were randomized into four groups: control (standard diet), HFD (high-fat diet), HFD+Orlistat (high-fat diet+orlistat), and HFD+WT (high-fat diet+white tea extract). A significant increase in malondialdehyde (MDA) levels and a decrease in total thiol (TT) levels were detected in the HFD group (*p* < 0.001). On the other hand, a decrease in the MDA level (*p* < 0.001) and an increase in the TT level were observed in the orlistat and white tea groups compared with those in the HFD group (*p* < 0.001). Histopathological examinations revealed that, compared with the HFD alone, orlistat and white tea reduced fat accumulation, prevented degenerative changes in hepatocytes, and decreased the histopathological damage score (*p* = 0.001). Immunohistochemical examinations of nuclear factor-kappa B (NF-kB/p65) revealed that compared with the HFD, orlistat and white tea reduced immunopositivity (*p* = 0.001). White tea decreases lipid peroxidation and oxidative stress. Both white tea and orlistat decreased fat formation and inflammation in the liver and regulated inflammation by reducing Nf-kB positivity. Nevertheless, further research is needed to assess their impact on human subjects.

## 1. Introduction

Nonalcoholic fatty liver disease (NAFLD), also known as metabolic dysfunction-associated steatotic liver disease (MASLD), is a metabolic condition characterized by excessive fat accumulation in hepatocytes as a result of impaired lipid metabolism in the liver. It is not associated with excessive alcohol consumption or other causes of fatty liver [1,2]. MASLD is the most common chronic liver disease, affecting approximately 30% of the total population globally [1]. Increasing obesity and unhealthy lifestyles are the most important factors that will make MASLD a widespread epidemic in the future [2]. Although lipid deposition in hepatocytes is considered a benign condition, it may cause inflammation by producing free oxygen radicals, i.e., reactive oxygen species (ROS) [3]. Inflammation can trigger a process that progresses to hepatocellular ballooning, fibrosis, cirrhosis, and hepatocellular cancer [1,4]. More importantly, the most common and most important cause of morbidity and mortality in MASLD is an increased risk of cardiometabolic diseases, not cirrhosis or hepatocellular cancer [1]. Despite extensive research, no effective treatment for MASLD exists aside from lifestyle modification [5].

Tea is one of the most consumed beverages worldwide after water [6]. Considering the content of catechins, the anti-inflammatory and antioxidant effects of tea have been investigated for many diseases [7]. The catechin with the greatest antioxidant effect in tea is epigallocatechin gallate (EGCG), which is mostly found in white tea [8]. Data in the literature indicate that tea may have positive effects on MASLD, including hepatic fibrosis [9,10,11].

High-fat diets (HFDs) cause hepatosteatosis [12]. Orlistat is a gastric and pancreatic lipase inhibitor that inhibits the absorption and digestion of dietary fat [13]. It is a Food and Drug Administration (FDA)-approved antiobesity medication [3]. In the literature, it has been shown that orlistat has curative and antiatherogenic effects on MASLD [14]. Although the positive effects of orlistat on MASLD have been attributed to its weight loss effect in most studies, data from the literature indicate that orlistat suppresses inflammation and improves endothelial dysfunction by inhibiting nuclear factor-kappa B (NF-κB) activation independent of weight loss [15]. However, orlistat has several side effects that limit its suitability, such as a decreased absorption of fat-soluble medications and vitamins, liver injury, appetite suppression, and headache [16].

The fact that more than 30% of the world’s population has MASLD, and that some of them are at risk of progressive liver and cardiometabolic diseases, has led to research into the development of therapeutic agents to treat MASLD [2]. However, no effective and safe agent has been approved for MASLD.

The consumption of white tea is generally safe and has very rare side effects [17]. Owing to a variety of bioactive compounds, such as polyphenols, alkaloids, polysaccharides, saponins, free amino acids, and pigments, white tea exerts hepatoprotective and cardiovascular-protective effects via anti-inflammatory, antioxidant, immunoregulatory, antidiabetic, antiobesity, and anticancer mechanisms [18,19,20,21,22,23,24].

This study aimed to discover fatty liver in rats subjected to a high-fat diet and examine the effects of orlistat and white tea on the liver. The antioxidant and anti-inflammatory properties of orlistat and white tea on liver tissue were examined by histopathological, immunohistochemical, and biochemical methods.

## 2. Materials and Methods

This experimental study was performed with approval from the Recep Tayyip Erdogan University Animal Research Ethics Committee (Rize, Turkey; date: 14 February 2023; approval number: 2023/12). The 32 male Sprague–Dawley rats (3–4 months) used in this study were obtained from Recep Tayyip Erdogan University, the Faculty of Medicine, Animal Research Unit. The rats were cared for under optimal laboratory conditions, which included 22–26 °C, 55–60% humidity, and a 12:12 h light/dark cycle according to the principles of the National Research Council’s Guide for the Care and Use of Laboratory Animals. The subjects were also allowed ad libitum access to water. All animal experiments were carried out in accordance with the UK Animals (Scientific Procedures) Act, 1986, and associated guidelines; the European Communities Council Directive of 24 November 1986 (86/609/EEC); and the National Institutes of Health Guide for the Care and Use of Laboratory Animals (NIH Publications No. 8023, revised 1978) [25].

### 2.1. Experimental Animals

Sprague–Dawley rats (male, n = 32, weight = 283 ± 26 g) aged 3–4 months were randomized into four equal groups. The quantity of animals in our study groups was established in accordance with the methodologies of Arifin, Charan, and Allgoewer et al. [26,27,28]. The rats in Group 1 (the control group) were fed only standard pellets (15–20 g/day/rat) for 12 weeks. In Group 2 (HFD), for 12 weeks, the rats were fed only a 15–20 g/day/rat high-fat diet. The rats in Group 3 (HFD+orlistat) were given a 15–20 mg/day/rat high-fat diet and 30 mg/kg/day orlistat by oral gavage for 12 weeks [29,30,31,32,33]. In Group 4 (HFD+WT), 5 mg/kg/day white tea extract was administered by oral gavage in addition to a 15–20 g/day/rat high-fat diet (HFD, 44% of energy comes from fat, Arden Research & Experiment) for 12 weeks [7,22,29,34,35,36]. After a 12 h fasting period and the completion of all the procedures, all the rats were sacrificed with 50 mg/kg ketamine HCl and 10 mg/kg xylazine, and the requisite liver tissue materials were removed [37].

### 2.2. Chemicals and Drugs

Ketamine hydrochloride (Ketalar, Pfizer İlaçları Ltd., Istanbul, Türkiye), xylazine hydrochloride (Rompun, Bayer, Istanbul, Türkiye), and orlistat (Thincal 120 mg/capsule, KOÇAK FARMA Ilac ve Kimya Sanayi A.S., Tekirdag, Türkiye) were used. All the chemicals used in the laboratory experiments were provided by Sigma Chemical Co. and Merck (Darmstadt, Germany).

### 2.3. Preparation of White Tea Extract

Camellia sinensis was obtained from CAYKUR A.S. (Rize, Turkey). The measured quantities of tea leaves designated for the research were weighed, combined with water, heated to 100 °C, allowed to cool somewhat (90–95 °C), covered, and steeped for 10 min [7]. The white tea produced daily was stored at room temperature and administered to the rats. The resulting infusion was cooled to room temperature and filtered through filter paper to be stored in dark bottles. The phenolic content, as analyzed with high-performance liquid chromatography (HPLC), of the WT, as determined by the Kacar B method of analysis, is listed in Table 1 [38,39].

### 2.4. Tissue Homogenization

A preparation of 20 mM 1 L sodium phosphate + 140 mM potassium chloride was made (pH 7.4). Then, 1 mL of homogenization solution was added to 100 mg of tissue, and the liver tissue was homogenized via a homogenizer (QIAGEN Tissue Lyser II) and centrifuged at 800× *g* for 10 min at 4 °C. Total thiol (TT) and thiobarbituric acid reactive substance (TBARS) assays were performed with the obtained supernatant [40].

### 2.5. Malondialdehyde (MDA) Analysis Procedure (TBARS Assay)

The TBARS assay was performed according to the methods of Ohkawa et al. [41]. A mixture of 200 µL of liver tissue supernatant, 50 µL of 8.1% SDS (sodium dodecyl sulfate, *w*/*v*), 375 µL of 20% acetic acid (*v*/*v*), and pH 3.5, and 375 µL of 0.8% thiobarbituric acid (TBA, *w*/*v*) was prepared. The mixture was strained, and the mixture was incubated in a boiling water bath for 1 h. After incubation, the mixture was cooled in ice water for 5 min and centrifuged at 750× *g* for 10 min. The resulting pink color was measured with a spectrophotometer at 532 nm. The results were calculated in nmol/g prt.

### 2.6. Total Thiol (TT) Analysis Procedure

Ellman’s reagent was used to determine the TT groups [42]. A total of 250 µL of liver supernatant was added to 1000 µL of 3 M Na2HPO4 and 250 µL of DTNB (DNTB, 5,5′-dithiobis (2-nitrobenzoic acid) (4 mg of DTNB prepared in 10 mL of 1% sodium citrate solution)), vortexed, and the absorbance at 412 nm was determined. The results are determined via a predetermined 1000 µM–62.5 µM reduced glutathione standard curve and are presented in nmol/g prt.

### 2.7. Histopathological Analyses

Liver tissue samples extracted from the rats were cut into 2 cm^3^ pieces and trimmed. The fixation process was performed by keeping the liver tissue samples in a 10% neutral formalin solution for 24 h. Histological tissue follow-up after fixation was performed with a tissue tracking device (Citadel 2000, ThermoScientific™, Cheshire, UK) via tissue tracking cassettes. Dehydration was performed by passing through the increasing ethanol series in the tissue tracking device. Afterward, the liver tissue samples were kept in two series of xylol solutions, and a clarification technique was performed. Finally, the paraffin inclusion process was performed by keeping the liver tissue samples in hard and soft paraffin. After the paraffin inclusion step, the liver tissue samples in the tissue embedding cassette removed from the tissue tracking device were blocked with hard paraffin via a paraffin blocking device (Leica EG 1150 H, Nussloch, Germany) and allowed to dry overnight. Serial sample sections of 4–5 µm thickness (Leica RM2255, Leica Biosystems, Nussloch, Germany) taken from the paraffin blocks were prepared on adhesive slides with a rotary microtome.

### 2.8. IHC Staining Protocol

After deparaffinization and endogenous peroxidase suppression, the sections were incubated with NF-kβ/p65 primary antibodies (ab16502, Abcam, Cambridge, UK) and secondary antibodies (ab205718, Abcam, Cambridge, UK) via an IHC/ISH device (Bond Max, Leica Biosystems, Melbourne, Australia) for 60 min. After incubation with primary and secondary antibodies, DAB was visualized with Chromagen (Ultraview, Leica Biosystems, Nussloch, Germany). Finally, the cell nuclei were counterstained with Harris hematoxylin.

### 2.9. Semiquantitative Analysis

The hepatic tissue sections of liver tissue stained with Harris hematoxylin and eosin G were evaluated for histopathological classification following the methodology employed in animal studies of nonalcoholic fatty liver (the scoring modification of Vansoun et al.). This assessment involved the examination of degenerative hepatocytes with lipid droplets, centrilobular degeneration of hepatocytes, perizonal degeneration of hepatocytes, and edematous regions, which were classified as absent = 0 (≤5%), mild = 1 (≤25%), moderate = 2 (≤50%), and severe = 3 > 50%) (Supplementary Material Table S1) [5,12,43]. For semiquantitative analyses, 20 different fields randomly selected from sections of each rat were scored via the ×40 objective by a histopathologist, who was blinded to the study groups, as follows: absent = 0 (≤5%), mild = 1 (≤25%), moderate = 2 (≤50%), and severe = 3 (>50%) (Supplementary Table S2).

### 2.10. Statistical Analysis

The quantitative and semiquantitative data obtained in our study were analyzed via the SPSS 20.00 (IBM Corp. IL, Chicago, IL, USA) statistical program. The Shapiro–Wilk test, Q–Q plot, skewness–kurtosis test, and Levene’s test were performed to determine whether the data were normally distributed. As a result of the biochemical analyses, we determined that the TBARS and total thiol tissue level data were parametric. Parametric data are presented as the means and standard deviations and were analyzed via one-way ANOVA. The differences in parametric data between groups were analyzed with the Bonferroni correction. Nonparametric data obtained by liver histopathological scoring and immune-positivity scoring are presented as the median and 25th–75th interquartile range, and differences between the groups are compared via the Kruskal–Wallis test and the Bonferroni-corrected Mann–Whitney U test, respectively. A *p* value less than 0.05 was considered statistically significant. However, owing to the exploratory nature of this study, the *p* value is only descriptive.

## 3. Results

### 3.1. Biochemical Results

#### 3.1.1. TBARS Results

TBARS was measured to investigate the effects of an atherogenic diet on membrane lipid peroxidation in liver tissue, and we found that the TBARS level was greater in the HFD group than in the control group (Table 2, *p* < 0.001). In contrast, we found that the TBARS level in liver tissue was lower in the orlistat treatment group than in the HFD group (Table 2, *p* < 0.001). Similarly, we observed that the TBARS level was significantly lower in the white tea-treated group than in the HFD group (Table 2, *p* < 0.001).

#### 3.1.2. Total Thiol (TT) Tissue Level Results

To investigate the effects of an atherogenic diet on antioxidant enzymes in liver tissue, we observed that the TT level was lower in the HFD group than in the control group (Table 2, *p* < 0.001). Total thiol levels were greater in the orlistat treatment group than in the HFD group (Table 2, *p* < 0.001). Similarly, we observed that the TT level was significantly greater in the white tea-treated group than in the HFD group (Table 2, *p* < 0.001).

### 3.2. Histopathological Results

**Figure 1:**
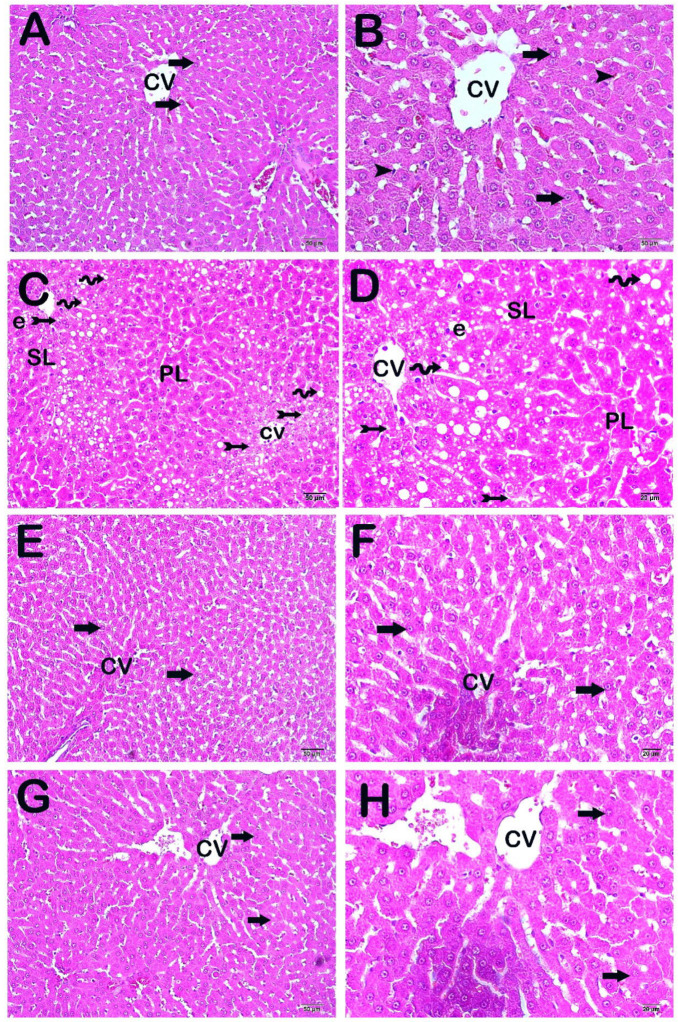
Representative light microscopy images of H&E-stained liver tissue; central vein (CV), centrilobular area (CL), and perilobular area (PL). (**A**) (×10), (**B**) (×40) Control group: remark cords (arrow), Kupffer cells (arrowhead), and sinusoids formed in normal hepatocytes (LPS: 0 (0–0)). (**C**) (×10), (**D**) (×40) HFD: Degenerative Remark cords consisting of degenerative hepatocytes (tailed arrow) containing diffuse fatty vacuoles (spirally arrow) are observed in the cytoplasm. Centrilobular (CL) and perilobular (PL) involvement, occasional edematous areas (LHDS median: 7 (7–9)). (**E**) (×10), (**F**) (×40) HFD+Orlistat: a decrease is observed in degenerative Remark cords, perilobular (PL) and centrilobular involvement, and edematous areas (LHDS median: 1 (0–3)). (**G**) (×10), (**H**) (×40) HFD+WT: Degenerative hepatocytes and edematous areas appear to be reduced. Centrilobular and perilobular involvement and degenerative Remark cords are reduced. (LHDS median: 0.5 (0–3)).

A light microscopy examination of liver sections from the control group revealed the presence of Remark cords consisting of normal hepatocytes (Figure 1A,B, Table 3, LHDS: 0 (0–0.5)). There were many necrotic hepatocytes with cytoplasmic vacuoles in the centrilobular and perilobular areas of the sections in the HFD group. However, diffuse edema was observed in the intralobular areas (Figure 1C,D, Table 3, LHDS: 7 (7–9)). A light microscopic examination of liver tissue from the orlistat treatment group revealed that the number of necrotic hepatocytes decreased in the centrilobular areas, especially in the perilobular areas. In addition, we observed that edematous areas decreased in the centrilobular and perilobular areas (Figure 1E,F, Table 3, LHDS: 1 (0–3)). Similarly, we found that necrotic hepatocytes and edematous areas decreased in the centrilobular and perilobular areas in the white tea application group (Figure 1G,H, Table 3, LHDS: 0.5 (0–3)).

### 3.3. Immunohistochemical Results

#### NF-kβ/p65 Primary Antibodies

**Figure 2:**
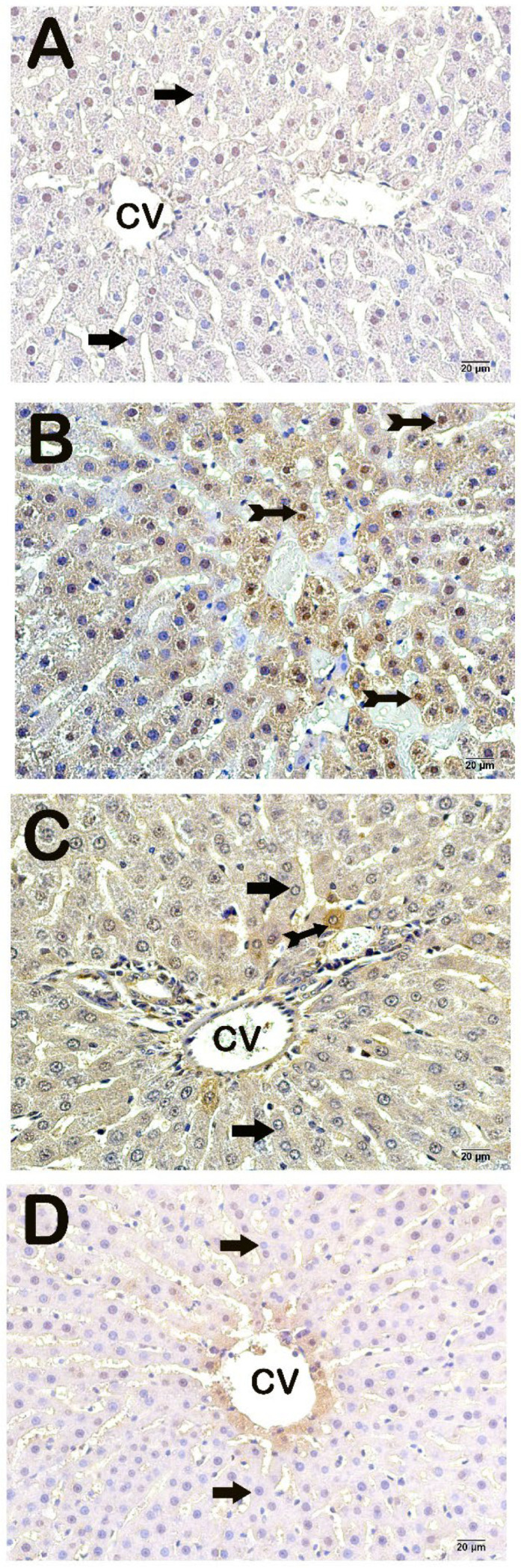
Representative light microscope images of sections of liver tissue incubated with NF-kβ/p65 primary antibodies. (**A**) (×40) Control group: immune-negative hepatocytes with a normal histological structure (NF-kβ/p65 positivity score: 0 (0–1)). (**B**) (×40) HFD group: dense NF-kβ/p65-positive hepatocytes (tailed arrow) are observed (NF-kβ/p65 positivity score: 2.5 (2–3)). (**C**) (×40): HFD+orlistat group: the number of cells positive for NF-kβ/p65 decreased (tailed arrow) in hepatocytes located in Remark cords in interlobular areas (NF-kβ/p65 positivity score: 1 (0–1)). (**D**) (×40): HFD+WT group: hepatocytes (arrow) show a decrease in NF-kβ/p65 positivity (NF-kβ/p65 positivity score: 0 (0–1)).

A light microscopic examination of liver tissue sections stained with an NF-kβ/p65 primary antibody revealed normal immune-negative cells among hepatocytes from the control group (Figure 2A, Table 4, *p* = 0.001; NF-kβ/p65 positivity score: 0 (0–0)). In contrast, an increase in the number of cells exhibiting intense NF-kβ/p65 positivity was observed in sections from the HFD group (Figure 2B, Table 4, *p* = 0.001, NF-kβ/p65 positivity score: 2 (2–2)). However, a decrease in the number of cells exhibiting NF-kβ/p65 positivity in hepatocytes from the HFD+Orlistat and HFD+WT groups compared with the HFD group was detected (Figure 2C,D, Table 4, *p* = 0.001; NF-kβ/p65 positivity score: 0 (0–1); NF-kβ/p65 positivity score: 1 (0–1)).

## 4. Discussion

This study covers changes in liver MDA (i.e., TBARS) and TT levels, histopathological changes, and Nf-kB-p65 immunohistochemical examinations of orlistat and white tea administration separately in rats fed a high-fat diet. Our findings suggest that both interventions individually have significant effects on liver health. First, orlistat administration significantly reduces MDA levels in the liver. Malondialdehyde is considered a marker of lipid peroxidation, and this reduction is consistent with the antioxidant properties of orlistat [44]. The lipase inhibitor activity of orlistat may also reduce lipid peroxidation by reducing fat absorption [3]. The increase in TT levels is another indicator of the antioxidant effects of orlistat. Glutathione is an important antioxidant that fights oxidative stress in cells [45]. The increase caused by orlistat may protect liver health by improving the cellular redox balance.

On the other hand, white tea application similarly contributes positively to liver health. White tea contains polyphenols that have antioxidant properties [17]. According to the findings of the present study, white tea increased TT levels and decreased MDA (i.e., TBARS) levels. The decrease in MDA levels and increase in TT levels may support the antioxidant effects of white tea. These findings suggest that the antioxidant capacity of white tea may protect liver cells against oxidative stress. Histopathological examinations revealed that fatty liver (steatosis) in high-fat diet-fed rats was alleviated after orlistat and white tea application. These findings indicate the potential for interventions to improve the morphology of the liver.

Nf-kB/p65 immunohistochemical studies helped us understand how orlistat and white tea affect inflammation. Our findings suggest that both interventions regulate the inflammatory response in the liver and inhibit Nf-kB-p65 activity. Nf-kB is a nuclear factor that is essential for regulating inflammatory responses [46]. An increase in the activity of this factor may contribute to the control of inflammation in the liver. These findings support the anti-inflammatory effects of orlistat. Similarly, white tea administration is associated with a significant decrease in Nfkb-p65 positivity. The antioxidant and anti-inflammatory properties of white tea may reduce inflammation in the liver by regulating Nf-kB activity. Studies in the literature highlight the complex relationships between Nf-kB and inflammation [46,47]. Situations in which Nf-kB is activated may increase inflammatory responses. Therefore, the positive effects of orlistat and white tea on Nfkb-p65 may reflect their potential to regulate inflammation in the liver.

Orlistat decreases fat absorption in the body primarily by inhibiting gastric lipase [32]. This process inhibits hepatic fat accumulation and may enhance liver health. The impact of orlistat on the liver is particularly evident in people with fatty liver disease since it diminishes hepatic fat accumulation and enhances liver function. In addition, orlistat can indirectly improve chronic subclinical inflammation associated with obesity through weight loss [48]. White tea, recognized for its elevated antioxidant levels, enhances liver function by mitigating the oxidative stress induced by free radicals [21]. White tea comprises biologically active constituents, including flavonoids and polyphenols, which confer a protective effect on the liver by mitigating inflammation and cellular damage [49]. Furthermore, the benefits of white tea, including a reduction in insulin resistance and the regulation of lipid metabolism, may also enhance liver functions [24]. MASLD is a multifactorial disease state that mechanically causes oxidative stress, insulin resistance, and excessive fat accumulation in the liver as a result of excessive fat consumption [47]. The hepatoprotective benefits of orlistat and white tea are attributed to distinct mechanisms. Orlistat immediately diminishes fat buildup by inhibiting fat absorption, but white tea protects the liver through its antioxidant benefits and anti-inflammatory characteristics [3,13,21,22,23,38,50,51,52,53]. A subsequent examination of these distinct processes in future research will increase our understanding of the roles these agents play in liver health.

Our study has several limitations. These limitations may affect the overall validity of our study and the interpretability of the results. Therefore, our study should be considered in this context. This study used an animal model involving rats fed a high-fat diet. However, animal models do not fully reflect human biology, and there may be differences in metabolism between humans and animals. More human-based research is needed to generalize the results directly to humans. The high-fat diet used in the present study represents high-fat nutrition in general. However, different fat compositions and dietary variations should also be examined, and the results may need to include these variations. Although orlistat and white tea were selected for specific targets, each of these substances has complex biological interactions. Therefore, the dosages and administration methods used in this study should be carefully evaluated and further optimized. This study examined the MDA and TT levels, lipid profiles, and immunohistochemical and histopathological changes in the liver. However, considering the complexity of the liver, a broader analysis of the metabolite profile and more parameters, such as gene expression, should also be performed. This study was conducted over a specific period, and longer-term studies are needed to evaluate long-term effects to assess whether the beneficial effects observed in MASLD persist after the discontinuation of treatment. Therefore, future long-term follow-up studies are needed to confirm our results and to confirm the potential of these interventions in human trials in the future.

One of the limitations of our study is the white tea application dose. White tea studies in the literature vary greatly in terms of the doses applied, and our study should be supported with different dosage applications in future studies [22,34,49,54,55]. Compared with other teas, white tea has the highest concentration of EGCG [6,23]. Despite its widespread promotion of putative health advantages, including anticancer characteristics, the absence of scientific data regarding the dosage levels of EGCG remains ambiguous [22]. Repeatedly high dosages of EGCG may induce hepatotoxicity and dyslipidemia [56]. A study has been conducted to ascertain the maximum tolerated nontoxic dose of EGCG and to discover the risk variables associated with therapy [56]. In a fourteen-day continuous therapy, two distinct administration methods were examined, providing enhanced [i.p. (intraperitoneal)] and restricted [p.o. (oral)] bioavailability. A trend toward dose- and route-dependent hepatotoxicity was noted, especially with intraperitoneal administration, and EGCG elevated the serum lipid profile concomitantly with hepatotoxicity. The acceptable fourteen-day dosage of EGCG was determined to be 21.1 mg/kg for intraperitoneal administration and 67.8 mg/kg for oral administration [56]. The severity of EGCG-induced toxicity is contingent upon the dosage, method of administration, and duration of treatment. In our HPLC analysis of white tea, the EGCG level was 9.2% dry matter. Our investigation employed the oral gavage method. Our research constitutes a pilot study with a duration of 12 weeks. Consequently, we selected the minimal dosage to prevent hepatotoxicity. Our subsequent investigations proceeded with increasing doses. Finally, Nf-kB immunohistochemical studies provide only limited information about the gene expression and functions of these proteins. Therefore, these findings should be supported by further molecular biology techniques and protein–protein interaction studies.

## 5. Conclusions

This study revealed beneficial effects on liver health in rats fed a high-fat diet when white tea and orlistat were administered. White tea decreases lipid peroxidation and oxidative stress. Both treatments decreased fat formation and inflammation in the liver and regulated inflammation by reducing Nf-kB positivity. Nevertheless, further research is needed to assess their impact on human subjects.

## Figures and Tables

**Table 1:** *Camellia sinensis* HPLC analysis results (specific catechins/total catechins).

-	%
Gallic acid	**2.87**
EGC	**0**
C	**0.93**
EC	**5.27**
EGCG	**74.68**
ECG	**16.25**
**Total catechins** **(EGC + C + EC + EGCG + ECG)**	**100**

EGC: Epigallocatechin, C: catechin, EC: Epicatechin, EGCG: Epigallocatechin-3 gallate, ECG: Epicatechin-3-gallate.

**Table 2:** Parameters of oxidative stress and lipid profile in the examined groups.

Parameters	Control (Group 1)	HFD (Group 2)	HFD+Orlistat (Group 3)	HFD+WT (Group 4)	*p*
**Total Thiols (nmol/g prt)**	744.72 ± 133.42	367.07 ± 93	850.62 ± 111.75	775 ± 194	<0.001 * 1–2 < 0.001 2–3 < 0.001 2–4 < 0.001
**TBARS (nmol/g prt)**	2.28 ± 2.53	4.19 ± 1.43	1.4 ± 0.81	2.41 ± 0.52	<0.001 * 1–2 < 0.001 * 1–3 < 0.001 * 2–3 = 0.004 * 3–4 = 0.004 * 2–4 = 0.048

Abbreviations: HFD, high-fat diet; WT, white tea; TBARS, thiobarbituric acid reactive substance. One-way ANOVA revealed that * *p* < 0.05 was significant. For pairwise comparisons, LSD post hoc * *p* < 0.05 was considered significant.

**Table 3:** Histopathological analysis scores (median ± 25–75% interquartile range).

Groups	Degenerative Hepatocytes (with Cytoplasmic Lipid Vacuoles)	Degenerative Centrilobular Remark Codons (Consist of Degenerative Hepatocytes)	Degenerative PeriLobular Remark Codons (Consist of Regenerative Hepatocytes)	Edematous Areas	LHDS
**Control**	0 (0–0.5)	0 (0–0)	0 (0–0)	0 (0–0)	0 (0–0.5)
**High-fat diet (HFD)**	2 (2–2) ^a^	2 (2–2) ^a^	1 (1–2) ^a^	2 (2–3) ^a^	7 (7–9) ^a^
**HFD+Orlistat**	0 (0–1) ^b^	0 (0–0.5) ^b^	0 (0–0.5) ^b^	0 (0–1) ^b^	1 (0–3) ^b^
**HFD+WT**	0 (0–1) ^b^	0 (0–1) ^b^	0 (0–1) ^b^	0 (0–1) ^b^	0.5 (0–3) ^b^

^a^ *p* = 0.001: compared to the control group, ^b^ *p* = 0.001: Compared to the HFD group, Kruskal–Wallis/Mann–Whitney U test with Bonferroni corrections.

**Table 4:** Immunohistochemical analysis results (median ± 25–75% interquartile range).

Group	NF-kβ/p65 Positivity Score
Control	0 (0–0)
High-fat diet (HFD)	2.5 (2–3) ^a^
HFD+Orlistat	1 (0–1) ^b^
HFD+WT	0 (0–1) ^b^

^a^ *p* = 0.001: compared to the control group, ^b^ *p* = 0.001: compared to the HFD group, Kruskal–Wallis/Mann–Whitney U test with Bonferroni corrections. Abbreviations: WT, white tea.

## Data Availability

All the data generated or analyzed during this study are included in this article. The data will be available upon reasonable request (contact person: filiz.mercantepe@saglik.gov.tr).

## Supplementary Materials

The following supporting information can be downloaded at: https://www.mdpi.com/article/10.3390/life14101283/s1. Table S1. Liver Histopathologic Damage Score (LHDS). Table S2. Immune-Positivity Score Method.
