# Supplementary material for: Investigating the Hepatic Response to Orlistat and White Tea in Rats on a High-Fat Diet
Source: Life (Basel). 2024 Oct 10;14(10):1283. doi: 10.3390/life14101283 (PMC11509274; doi:10.3390/life14101283)
Supplement: Supplementary file 1 [file life-14-01283-s001.zip › life-3232139-supplementary.pdf]

Table S1. Liver Histopathologic Damage Score (LHDS).

Score	**Findings**
Degenerative hepatocytes with lipid droplets	
0	≤5%
1	≤25%
2	≤50%
3	>50%
Centrilobular involvement with degenerative hepatocytes	
0	≤5%
1	≤25%
2	≤50%
3	>50%
Perizonal involvement with degenerative hepatocytes	
0	≤5%
1	≤25%
2	≤50%
3	>50%
Edematous Areas	
0	≤5%
1	≤25%
2	≤50%
3	>50%

Table S2. Immune-Positivity Score Method.

Score	Findings
1	≤5%
2	≤25%
3	≤50%
0	>50%
